# Development and preliminary validity of an Indonesian mobile application for a balanced and sustainable diet for obesity management

**DOI:** 10.1186/s12889-022-13579-x

**Published:** 2022-06-20

**Authors:** Rina Agustina, Eka Febriyanti, Melyarna Putri, Meriza Martineta, Novi S. Hardiany, Dyah E. Mustikawati, Hanifa Hanifa, Anuraj H. Shankar

**Affiliations:** 1 Department of Nutrition, Faculty of Medicine, Universitas Indonesia – Dr. Cipto Mangunkusumo General Hospital, Jl, Salemba Raya no 6, Jakarta, Indonesia 10430; 2 Human Nutrition Research Center, Indonesian Medical Education and Research Institute (HNRC-IMERI), Faculty of Medicine, Universitas Indonesia, Jakarta, Indonesia; 3 Department of Nutrition Faculty of Medicine, Universitas Muhammadiyah Sumatera Utara, Medan, Indonesia; 4 Department of Nutrition, Faculty of Medicine, Universitas Sumatera Utara, Medan, Indonesia; 5 Department of Biochemistry & Molecular Biology, Faculty of Medicine, Universitas Indonesia, Jakarta, Indonesia; 6 Ministry of Health Republic of Indonesia, Jakarta, Indonesia; 7 Centre for Tropical Medicine and Global Health, Nuffield Department of Medicine, University of Oxford, Oxford, UK; 8 Eijkman-Oxford Clinical Research Unit, Eijkman Institute for Molecular Biology, Jakarta, Indonesia

**Keywords:** Mobile apps, Balanced diet, Sustainable diet, Formative research, User acceptance test, Jakarta

## Abstract

**Background:**

Mobile applications such as personalized tracking tools and food choice aids may enhance weight loss programs. We developed and assessed client preferences for the content, user interface, graphics, and logic flow of a mobile application, and evaluated its validity for tracking compliance with weight control and making healthy and sustainable food choices.

**Methods:**

Our four-stage study comprised formative research, application development, acceptance assessment, and validity. The formative research included literature reviews and six focus groups with 39 respondents aged 19–64 years at high risk for obesity. The development stage included programmer selection, defining application specifications, design, and user interface. Prototype acceptability was assessed with 53 respondents who graded 17 features of content, graphic design, and application flow (ranked as good, moderate, and poor). A feature was considered to have "good" acceptance if its mean response was higher than the mean of overall responses. The validity was assessed in 30 obese women using Bland–Altman plots to compare results from dietary intake assessment from the application to conventional paper-based methods.

**Results:**

The application was named as EatsUp®. The focus group participants defined the key requirements of this app as being informative, easy, and exciting to use. The EatsUp® core features consisted of simple menu recommendations, health news, notifications, a food database, estimated portion sizes, and food pictures. The prototype had a "good" overall acceptance regarding content, graphics, and flow**.** Fourteen out of 17 parameters were graded as "good" from > 70% of respondents. There was no significant difference between the rated proportions for content, graphics, and app flow (Kolmogorov–Smirnov Z-test, *p* > .05). The agreement using the Bland–Altman plots between EatsUp® and the paper-based method of measuring food intake was good, with a mean difference of energy intake of only 2.63 ± 28.4 kcal/day (*p* > 0.05), well within the 95% confidence interval for agreement.

**Conclusions:**

The EatsUp® mobile application had good acceptance for graphics and app flow. This application can support the monitoring of balanced and sustainable dietary practice by providing nutritional data, and is comparable with conventional dietary assessment tools, and performed well in tracking energy, macronutrient, and selected micronutrients intakes.

**Trial registration:**

NCT03469869. The registration date was March 19, 2018.

## Introduction

Obesity is the fifth leading cause of mortality worldwide, with at least 2.8 million deaths each year from direct and indirect complications [1, 2]. Globally there are 650 million obese adults, a prevalence of 13%, which has nearly tripled between 1975 and 2016 [3]. In Indonesia, obesity prevalence has steadily increased from 2007–2018, from 10.5% to 21.8% [4].

The treatments for obesity include dietary modifications, physical exercise, pharmacological interventions, and surgery [5–7]. A restricted balanced diet is a standard recommendation for people with obesity [8]. A sustainable diet would be both healthy and protective of biodiversity and planetary health, and would meet nutritional needs and environmental impact requirements [9–14]. The working group of the UK's Green Food Project, set up by the Department for the Environment, Food and Rural Affairs, has produced principles and guidelines for a sustainable diet: consumption of more plant-based foods, the inclusion of at least five portions of fruits and vegetables per day, valuing food by asking where it comes from and how it is produced, moderation in meat consumption, enjoying more peas, beans, nuts, and other sources of plant-based protein, choosing fish from sustainable stocks, the inclusion of milk and dairy products while seeking plant-based alternatives including those fortified with additional vitamins and minerals, drinking tap water, and eating fewer foods high in fat, sugar, and salt [9], as was also outlined by the EAT-Lancet Commission on a healthy diets from sustainable food systems [15].

The main challenge in obesity management is maintaining compliance with weight loss programs. Mobile applications (apps) are tools that may potentially enhance short- and long-term compliance with weight reduction programs [16, 17]. Self-monitoring with apps may enable individuals to become aware and capable of identifying success or failure in meeting set goals. Indeed, self-monitoring of behaviors during weight management programs has been shown to facilitate adherence and lead to increased effectiveness [18]. Increased monitoring has been associated with greater efficacy for weight loss. In fact, weight measurement every one to three days has been shown to improve weight loss [19].

In Indonesia, 67.2% of the population (more than 183 million people) currently use smartphones with internet connections [20]. While there are many smartphone apps for dietary tracking and meal management, few available apps provide menus with Indonesian foods, and those that exist are still very limited. Therefore, the study aimed to design, deploy, validate and assess a mobile app that included the appropriate content, graphic design, and logic flow to enhance compliance with weight control regimens and selecting healthy and sustainable foods. We report herein the acceptance of a mobile app's contents, features, and design for a balanced and sustainable diet.

## Methods

### Design and setting

The study had four stages: formative research, app development, assessment of acceptance, and a validity test. In the formative research stage, qualitative methods were employed to obtain information from users to conceptualize the app's development. The development stage included selecting a developer and programming language, and building the app design. The acceptance stage assessed the initial development and aimed to improve the app's performance. The validity stage compared compliance tracking with the app to tracking with conventional paper records. The study was approved by the Health Research Ethics Committee of the Faculty of Medicine Universitas Indonesia – Dr. Cipto Mangunkusumo General Hospital (FMUI–RSCM) with ethical number 829/UN2.F1/ETIK/2017.

### Procedures

Formative research was critical for developing the app and consisted of two phases: a literature review and focus group discussions. The literature review identified the relevant evidence-based theories on innovative obesity management programs, and potential gaps in current interventions (Table 1). A crucial step in developing the app was to systematically examine previous research about weight loss guidelines for people with obesity, the existence of balanced and sustainable diet programs, and other available dietary mobile apps. This identified methods by which weight loss guidelines had been applied and how effective they were as short- to medium- or long-term behavioral change strategies. Subsequently, previous studies were translated into technical requirements, and used by the information technology specialists to guide app development. This meant the app's technical features (e.g., push notifications and the user interface) and the tailored interactive content were all rooted in an evidence- and theory-based framework of a sustainable diet and weight loss guidelines for people with obesity.

**Table 1:** Key points from the literature review on the management of obesity and targeted weight loss

Author	Design	Results
Garvey WT et al., 2016 [21]	AACE/ACE Guidelines	Obesity management consisted of restriction of calories, lifestyle/behavioral therapy, pharmacotherapy, and surgery
Pagoto et al., 2013 [22]	Cross sectional	A weight loss mobile app with behavioral strategies was an effective, evidence-based weight loss intervention
Gilliland et al., 2015 [23]	RCT	A smart appetite app was effective in improving the awareness and consumption of healthy foods
Lin L et al., 2015 [24]	Literature review	The human-chatbot interaction enhanced the process of data collection
Geethanjali et al., 2017 [25]	Systematic review	Chatbot development was still in its infancy and many techniques are yet to be discovered
Harray et al., 2015 [26]	RCT	Using mobile food records to assess dietary adherence is a novel and innovative approach
Schoenaker DA et al., 2016 [27]	Systematic review	The recommended balanced diet limited to the saturated fats and cholesterol consumptions
Kramer et al., 2017 [28]	Cross Sectional	Reducing meat consumption was effective in reducing the dietary environmental impact

*RCT* randomized clinical trial

Papers published between 2013 and 2017 from several databases, including PubMed, NCBI, ScienceDirect, Elsevier, Plos One, and Biomed Central, were reviewed. The keywords used were a combinations of "balanced and sustainable diet", "obesity", "weight loss", and "mobile apps". In the next step of the formative research, the app's feasibility was tested by organizing the focus group discussions (FGDs). Subjects were recruited in September 2017 and consisted of 39 healthy government employees comprising 30 males and nine females aged 19–64 years who lived in Jakarta, Indonesia, and owned Android smartphones. Participants were divided into six groups (3–5 people in each group) based on their education levels (senior secondary school and graduate), dietary habits (balanced diet or not), and physical activity as very active or not (i.e. sedentary, low activity and active) [29–31]. Each group was asked about their sociodemographic characteristics such as age, sex, and occupation and their prior knowledge of balanced and sustainable diets, history of using food records, history of using dietary mobile apps, and the kind of app that they expected would be useful.

The second stage of this study included developing the mobile app for the Android platform by collaborating with information technology application developers, graphic designers, communication experts, physicians, and dietitians. The app was developed using Android Studio IDE and the Java programming language. Backend support and data processing used web-based server systems, and MySQL was used as the database management system. The app was compatible with Android version 4.4 (KitKat) and above. Physicians identified the total calories, carbohydrates, proteins, fats, and other micronutrients needed by each user, while the dietitians helped to make menu recommendations. The total caloric needs were calculated based on the Harris-Benedict Equation [32]. Hence, the macronutrient requirements were divided into 55% carbohydrate, 20% protein, and 25% fat. Using these features, the app provided users with guidance on attaining a balanced and sustainable diet and increased awareness of staying healthy throughout their dietary programs. The app could guide people to achieve weight loss by suggesting adjusted menu recommendations, monitoring habitual and actual intakes (calories, macronutrients, and micronutrients), physical activity, and greenhouse gas emission (GHGE). The app enabled people to track their targets for losing weight, body mass index (BMI), waist circumference, and body compositions (i.e., fat mass and fat-free mass). Also, the app helped users to monitor their insulin resistance indicators (fasting glucose, fasting insulin, HOMA IR, HbA1C), lipid profiles, uric acid levels, and inflammation markers, such as TNFα and hsCRP. Additionally, the app facilitated communication with physicians to ask questions about intake profiles, health conditions, or other nutrition and health concerns.

The third stage was the assessment of acceptance of the app, which was named EatsUp*®*. Subjects were recruited in January 2018 and consisted of 19 healthy males and 34 healthy females aged 19–64 years who lived in Jakarta, Indonesia, and had the willingness to join the study, and owned Android smartphones. Subjects with interest and consenting to this study were mostly females. This is in line with a study conducted by Mohajer, et al. (2019) that reported study cooperation of female participants tended to exceed that of males [33].

A minimum sample size of 53 subjects was calculated based on previous studies that were analogous to the formative research (stage one) of this study. The results of the acceptability study are described in Table 4 [34]. Each subject installed EatsUp*®* on their Android smartphone and was shown how to use it. Sociodemographic information, including age, sex, and occupation, was recorded. EatsUp*®* allowed users to input their weight, height, and date of birth, which were then used automatically to calculate BMI, nutritional status classification, total energy expenditure, and dietary macronutrient composition. The app permitted users to enter specific types of foods or beverages and the respective dietary data, and this information was later used to build the dietary record. After one to two days, the subjects were interviewed regarding their acceptance of EatsUp*®* in terms of contents, design, and logic flow. They were also asked to comment on the limitations and advantages and suggest future improvements.

A validity test for EatsUp*®*was performed in the fourth stage. Subjects comprised 30 overweight or obese women aged 19–59 years with BMI ≥ 25.0 kg/m2 who had Android smartphones [35]. This sample size was determined based on a rule of thumb [36], and in our study yielded a power of 48% to discern a difference of 26.4% for mean difference of energy intake between a paper-based method and the app. Subjects underwent anthropometry assessments for body weight and height, and dietary assessments using a paper-based 2 × 24-h food recall method. Subjects were asked to use EatsUp*®* for a week, starting with the personal data registration process, input of individual body weight and height for the nutritional status and individual total energy requirement calculation, and inputting all food and drinks consumed.

### Data analysis

Qualitative analysis was performed to describe the formative research using matrix analysis. A descriptive analysis was performed to illustrate the characteristics of the subjects and the acceptance of the app. The subjects' characteristics and the 17 key assessment responses were described in frequency and percentage as indicated in Table 2. Three rankings were used to describe EatsUp*®* based on subjects' preferences: good, moderate, and poor. The statistical significance of the differences between statements with different rankings was measured using the Kolmogorov–Smirnov Z-test. The validation test was measured using a one-sample T-test, and the Bland–Altman plot defined the agreement for both methods.

**Table 2:** Characteristics of subjects in the formative research, acceptance, and validity testing stages of the application

Variables	Formative research (*n* = 39)	Acceptance test (*n* = 53)	Validity test (*n* = 30)
Sex, n (%)			
Male	30 (77)	19 (36)	0 (0)
Female	9 (23)	34 (64)	30 (100)
Age (years), n (%)			33.5 ± 10.0
19–30	19 (49)	41 (77)	
31–64	20 (51)	12 (23)	
Occupation, n (%)			
Employee		41 (77)	10 (33.3)
Non-employee		12 (23)	20 (66.6)
Education level, n (%)			
Senior secondary school			6 (20.0)
Graduate			24 (80.0)
Months of owning an Android smartphone, n (%)			
< 6		0 (0)	
≥ 6		30 (100)	
Anthropometric assessment, m ± SD			
Height (cm)			154 ± 3.7
Weight (kg)			71.8 ± 9.8
BMI (kg/m^2^)			30.2 ± 3.8
Nutritional status, n (%)			
Obesity I			14 (46.7)
Obesity II			16 (53.3)

*BMI* Body mass index

## Results

From the matrix analysis of FGD results on Table 3, some participants lacked knowledge about a balanced and sustainable diet, 80% did not realize they were practicing it, and 85% had never known about food records, but 45% knew about existing dietary mobile apps and 70% had not used them. Participants stated they needed an easy, simple, attractive, informative, and user-friendly app that could monitor calorie intake and dietary composition and provide nutrition facts, menu recommendations, and energy output from physical activity.

**Table 3:** Topics and statements based on a matrix analysis of the focus group discussions among Indonesian adults

Topic	Statement or Quotation from the adults
Understanding balanced and sustainable diets	A balanced diet is classified as '*4 sehat 5 sempurna*' (4 health, 5 perfect). A sustainable diet is a diet containing organic food
Practicing a balanced and sustainable diet	Not yet, just planned but not realized
Understanding of food records	Do not know about it
Easy, daily food records	Easy but troublesome
Keeping a food record with paper-based, web-based, or mobile apps	Preferably using mobile apps because they are easy to use, efficient, and can be taken anywhere
Using previous dietary mobile apps	Knowledge of them without using them
Helpful features	Monitoring calorie intake and food composition, nutrition facts, menu recommendations, and energy expenditure from physical activity
Level of boredom using a dietary mobile app	It depends on features and input frequency
Expectations of a mobile app	Easy to use, simple, attractive, informative, and user-friendly

In the development stage, pictures of food, detailed food items (name of food, portion size, and nutrient content), and menu recommendations were uploaded into the EatsUp*®*database. Detailed food items were uploaded from the NutriSurvey database that was based on Indonesian food composition tables [37].

Menu recommendations and pictures of foods were based on balanced and sustainable diets and uploaded to the server database. Calculations of the BMI and total energy expenditure were based on the Harris-Benedict equation, and programmed to provide nutritional status and total energy expenditure for each individual. EatsUp*®* had a push notification reminder to alert the user of deviations from the recommendations (calories, macronutrient composition, water, and GHGE).

**Fig. 1:**
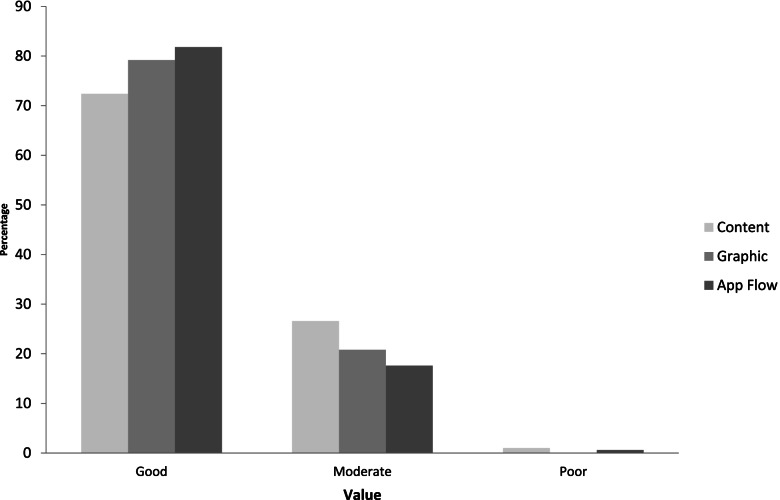
The proportion of rankings of statements from the subjects (*n* = 53)

Table 4 indicates that the app was well-accepted regarding content, graphics, and logic flow. However, some parameters scored below 70%, such as understanding of terminology (57%), ability to monitor the goal (68%), and easy-to-follow menu recommendations (62%). Based on the Kolmogorov–Smirnov Z test, there was no significant difference between the proportions for acceptance of content, graphics, and app flow (*p* > 0.05), as indicated in Fig. 1.

**Table 4:** Acceptance of the mobile application by adults in Jakarta (*n* = 53)

Parameters	Good n (%)	Moderate n (%)	Poor n (%)
Content			
Understanding of information	38 (72)	15 (28)	0 (0)
Understanding of terminology	30 (57)	22 (42)	1 (2)
Appropriate food pictures	38 (72)	14 (26)	1 (2)
Appropriate information	40 (75)	13 (25)	0 (0)
Ability to monitor the goal	36 (68)	16 (30)	1 (2)
Know caloric intake and the composition	42 (79)	11 (21)	0 (0)
Know nutrition facts	42 (79)	11 (21)	0 (0)
Easy to follow the menu recommendations	33 (62)	19 (36)	1 (2)
Can communicate with a doctor	37 (70)	16 (30)	0 (0)
Useful reminders	44 (83)	8 (15)	1 (2)
Useful information	41 (77)	11 (21)	1 (2)
Graphics			
Attractive color combination	40 (75)	13 (25)	0 (0)
Appropriate font size	44 (83)	9 (17)	0 (0)
Attractive appearance	41 (77)	12 (23)	0 (0)
App flow			
Understanding of commands	41 (77)	12 (23)	0 (0)
Easy to input food and beverages	43 (81)	9 (17)	1 (2)
Easy to track the caloric intake and its composition	46 (87)	7 (13)	0 (0)

**Fig. 2:**
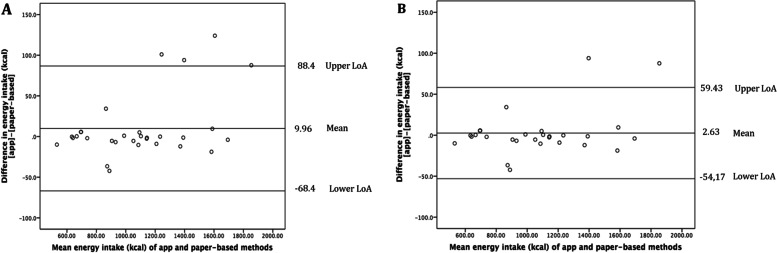
Agreement between methods (apps versus paper-based calculation) for energy intake using the Bland–Altman plot **A**. Without excluding outliers (*n* = 30) and **B**. After excluding outliers (*n* = 28); LoA, limit of agreement

The mean difference for energy intake between the EatsUp*®* app and the paper-based method was not significant (9.96 ± 39.2 kcal/day without excluding outliers; and 2.63 ± 28.4 kcal/day after excluding outliers, *p* > 0.05). The lower and upper limits of agreement (LOA) between energy intake methods ranged from -8.4 to 24.9 kcal/day. According to Figs. 2, A, and B, the Bland–Altman plots for energy intakes demonstrated that data for 30 subjects were within the 95% LOA, albeit with a few outliers.

## Discussion

Our formative research and preliminary results herein show that Indonesian adults could define a dietary mobile app's desired qualities such as being easy, informative, and exciting to use. In the development process, EatsUp*®* was engineered to record dietary data, give users menu recommendations based on their caloric targets, and include an evaluation of all biomarkers linked to complications of obesity for obese individuals. Finally, the acceptance test of EatsUp*®* showed that although it could be used as part of an obesity management regimen, it still needed improvement.

According to Duff et al., the app's technical aspects, such as push notifications and the user interface, and the interactive content, should be based on evidence and a theoretical framework of health behavior change. Developing an app-based health behavior change should consist of four key stages, 1) systematic review, 2) app development, 3) feasibility and acceptability testing of the prototype, and 4) evaluation and implementation [38]. A good app for managing obesity should have a feature for self-monitoring, a series of programs for weight loss, and strategies for changing lifestyle behaviors such as improvement of motivation, stress management, and problem-solving assistance [22]. Our app aimed to meet these requirements, and additional specific ones from users we assessed. The qualitative findings and randomized clinical trial (RCT) conducted by Mummah et al. showed that people need a mobile app that is accountable, efficient, and easy to use for dietary self-monitoring. Also, the apps should have reminder notifications, simple features, weekly reports, and features for planning meals in advance [39]. These findings were similar to those from our formative research study. It can thus be concluded that the app should be easy to use for diverse populations, provide health news, push notifications, a food database, estimated portion sizes, and food images to facilitate compliance with long-term dietary interventions.

Schoffman et al. reported that essential health content and concrete recommendations in an app required collaboration between diverse experts [40]. Ananda et al. stated that a mobile app might be an innovative tool to facilitate individual health behavior change interventions [41]. One limitation of this study was the lack of a complete Indonesian food composition table to estimate the nutrient intake. All food databases in the application were from the 2007 NutriSurvey food database, and hence, were not up-to-date.

The app was found to have a good overall acceptance among users regarding its graphics and app flow. Nevertheless, the content still needed improvement, especially in making sure that users understood the terminologies and that menu recommendations were easy to follow. Smith et al. indicated that web and mobile phone apps have advantages over standard face-to-face programs, such as 24/7 availability, less burden, high acceptability in target populations, greater program adherence, lower costs, the possibility of self-monitoring, and the ability to reach a large target population [42]. In addition, Ansa et al. stated that health promotion interventions should be culturally appropriate and tailored to meet the needs of the target population [43].

The Bland–Altman plots showed a good level of agreement between EatsUp*®* and the paper-based dietary tracking approach; and for a range of intakes most of the data points were located within the LOA. This suggested the mobile app was able to estimate individual intakes accurately. This is consistent with the results of Ahmed et al. [44], who reported that a tablet app was comparable to the traditional dietary assessment method. Similar to the findings of Timon et al. [45], the feasibility of computer-based dietary assessment was comparable to that of a 4-day estimated food diary.

The EatsUp*®* app had some limitations. It required a live internet connection and was designed for only the Android platform. However, respondents found the app useful, easy to carry as it is part of a smartphone, and easy to use because of its paperless food records, attractive design, use of images of foods, and attractive color schemes. The EatsUp*®*, therefore, had useful features and good potential for usability but needed to be improved for optimal use. The other limitations of this study were the relatively small sample size, and that we did not assess app users below the level of senior secondary school. While the ease-to-use features in the app may be suitable for lower educated individuals [39], further research is needed to verify this.

## Conclusion

In conclusion, this preliminary study of the EatsUp® app suggests it may increase adherence to an obesity management program. Further studies would be warranted for EatsUp® and similar apps. The app had good acceptance regarding its graphics and app flow. The balanced and sustainable dietary mobile app with added nutritional data was comparable to conventional dietary assessment methods, and performed well in assessing energy, macronutrient, and selected micronutrient intakes. The future improvement of EatsUp*®* might include resolving technical problems such as minor bugs, addition of offline capability and expansion to other platforms such as iOS and migration to the Fast Healthcare Interoperability Resources (FHIR) HL7 standard to enhance its interoperability. EatsUp® could be used for further research concerning intervention tools for obese persons.

## Data Availability

All of the material is owned by the authors and/or no permissions from the third party are required. The datasets used and/or analyzed during the current study are available from the corresponding author.
